# Metforminium Decavanadate as a Potential Metallopharmaceutical Drug for the Treatment of Diabetes Mellitus

**DOI:** 10.1155/2016/6058705

**Published:** 2016-03-28

**Authors:** Samuel Treviño, Denisse Velázquez-Vázquez, Eduardo Sánchez-Lara, Alfonso Diaz-Fonseca, José Ángel Flores-Hernandez, Aarón Pérez-Benítez, Eduardo Brambila-Colombres, Enrique González-Vergara

**Affiliations:** ^1^Laboratorio de Investigación en Química Clínica, Facultad de Ciencias Químicas, Benemérita Universidad Autónoma de Puebla, 18 Sur y Avenida San Claudio, Colonia San Manuel, 72570 Puebla, PUE, Mexico; ^2^Departamento de Bioquímica Clínica, Facultad de Ciencias Químicas, Benemérita Universidad Autónoma de Puebla, 18 Sur y Avenida San Claudio, Colonia San Manuel, 72570 Puebla, PUE, Mexico; ^3^Laboratorio de Bioinorgánica Aplicada, Centro de Química ICUAP, Benemérita Universidad Autónoma de Puebla, 18 Sur y Avenida San Claudio, Colonia San Manuel, 72570 Puebla, PUE, Mexico; ^4^Departamento de Farmacia, Facultad de Ciencias Químicas, Benemérita Universidad Autónoma de Puebla, 18 Sur y Avenida San Claudio, Colonia San Manuel, 72570 Puebla, PUE, Mexico; ^5^Laboratorio de Nuevos Materiales, Facultad de Ciencias Químicas, Benemérita Universidad Autónoma de Puebla, 18 Sur y Avenida San Claudio, Colonia San Manuel, 72570 Puebla, PUE, Mexico

## Abstract

New potential drugs based on vanadium are being developed as possible treatments for diabetes mellitus (DM) and its complications. In this regard, our working group developed metforminium decavanadate (MetfDeca), a compound with hypoglycemic and hypolipidemic properties. MetfDeca was evaluated in models of type 1 and type 2 diabetes mellitus, on male Wistar rats. Alloxan-induction was employed to produce DM1 model, while a hypercaloric-diet was employed to generate DM2 model. Two-month treatments with 3.7 *μ*g (2.5 *μ*M)/300 g/twice a week for DM2 and 7.18 *μ*g (4.8 *μ*M)/300 g/twice a week for DM1 of MetfDeca, respectively, were administered. The resulting pharmacological data showed nontoxicological effects on liver and kidney. At the same time, MetfDeca showed an improvement of carbohydrates and lipids in tissues and serum. MetfDeca treatment was better than the monotherapies with metformin for DM2 and insulin for DM1. Additionally, MetfDeca showed a protective effect on pancreatic beta cells of DM1 rats, suggesting a possible regeneration of these cells, since they recovered their insulin levels. Therefore, MetfDeca could be considered not only as an insulin-mimetic agent, but also as an insulin-enhancing agent. Efforts to elucidate the mechanism of action of this compound are now in progress.

## 1. Introduction

The use of metal-based drugs as therapeutic agents dates back to ancient cultures who lived in Mesopotamia, India, Egypt, and China, as early as 2500 B.C. [1]. Making a great leap in the history of metals in medicine up to Renaissance era, it is important to mention the work of Paracelsus and his concept of doses-response, coming from the introduction of mercury, arsenic, tin, lead, and antimony (among others) into the* Materia medica*: “All substances are poisons: there is none which is not a poison. The right dose differentiates a poison and a remedy” [2]. For this reason, the deep understanding about chemistry of metals within biological systems is very important.

Currently, the metal-based drugs are being used for the treatment of a variety of pathologies such as diabetes, cancer, rheumatoid arthritis, and inflammatory and cardiovascular diseases [3–5]. The health status of a person and physiological disorders and diseases are usually related to the presence or absence of metal ions and/or their corresponding complexes formed with biomolecules in the body. Thus, the metals called trace elements are necessary for many biological processes. In this sense, vanadium is a biometal capable of improving the metabolism of lipids and carbohydrates, as it has been reported for some of their compounds.

Despite the fact that the physiological pathways are unclear, it is now known that vanadate has different pharmacokinetics compared to the decavanadate and therefore different pharmacological activities, although, in relation with the carbohydrate and lipid metabolism, these act very similar. Although the vanadium intracellular concentration (+5, vanadate) is very low for the decavanadate species to be formed, it has been described that V10 was formed in acidic compartments in* Saccharomyces cerevisiae *that were grown in media containing vanadate. Thus it has been proposed that once it is formed the rate of decavanadate decomposition is slow (half-life time of hours) enough to allow observing its effects not only in vitro, but also in vivo [6–10].

Under these considerations the metallopharmaceuticals based on vanadium with potential hypoglycemic features could be considered as an admissible alternative for the treatment of diseases involving glycemic control, both in patients being insulin-requiring and insulin-independent diabetes mellitus types 1 (DM1) and 2 (DM2), respectively, and in patients with other diseases that have in common carbohydrate and lipid disorders [11].

Actually, diabetes mellitus represents a global public health problem. It has been estimated that 347 millions of people have diabetes mellitus until 2014. This pathology is defined as a metabolic disorder characterized by chronic hyperglycemia with disturbances in the metabolism of carbohydrates, lipids, and proteins resulting from defects in insulin secretion, its action, or both. Additionally, it is worth mentioning that DM represents the end stage of a chronic and progressive heterogeneous syndrome characterized by a series of metabolic disorders (dysglycemia and dyslipidemia), promoted by environmental factors, genetic susceptibility, insulin resistance, and a decrease in hormone secretion by pancreatic *β* cells [12, 13]. This pathology is a major cause of morbidity and mortality around the world, including Mexico [14].

**Figure 1:**
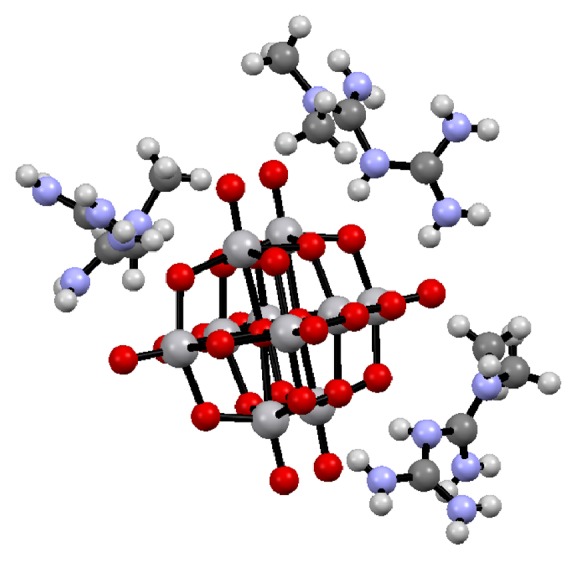
Ball and stick representation of metforminium decavanadate (H_2_Metf)_3_[V_10_O_28_]·8H_2_O. Water molecules are omitted for clarity [11].

Most patients diagnosed with DM2 should begin their therapy with lifestyle changes (lifestyle counseling, weight-loss education, exercise, etc.). When these lifestyles are not enough to achieve or maintain the glycemic levels, metformin monotherapy should be added soon after diagnosis, unless there are contraindications or intolerance. Metformin possesses a long list of evidences detailing its efficacy and safety; moreover it is very inexpensive and may reduce risk of cardiovascular events [15]. It has also been reported that metformin consumption has some disadvantages, such as gastrointestinal side effects (diarrhea, abdominal cramping), risk of lactic acidosis (rare), vitamin B12 deficiency, acid reflux, chronic kidney disease, hypoxia, and dehydration [16–18]. Additionally, we have reported that chronic administration of metformin showed multiorgan complications in the development of steatosis. By contrast, metforminium decavanadate (MetfDeca) presented in Figure 1 showed an improvement in the intracellular biochemical behavior and also a recovering of the adequate levels of lipids and carbohydrates [11].

Therefore, in the research of the vanadium-based metallopharmaceuticals both their beneficial physiological function and their potential toxicity should be considered [10, 19]. For these reasons, in this paper we focused on the role of MetfDeca as a potential new drug for the treatment of DM (and related disorders) and its toxicity effects in rat models of hyperglycemia, both requesting insulin and insulin-independent, since they are altered in the metabolism of carbohydrates and lipids.

## 2. Material and Methods

Male Wistar rats (70–100 g) were provided by the Bioterium “Claude Bernard,” of the Benemérita Universidad Autónoma de Puebla. The rats were housed in a climate-controlled and light-regulated facility with 12/12 h day/night cycles with free access to food and water “ad libitum.” All procedures described in this study are in accordance with the Guide for the Care and Use of Laboratory Animals of the Mexican Council for Animal Care NOM-062-ZOO-1999. Every effort was made to minimize the number of animals used and to ensure the minimal animal pain and/or discomfort. The animals were conditioned with normal calorie diet by 15 days. The diet used was 5001 of the LabDiet (Laboratory Rodent Diet); its composition can be consulted on the manufacturer's website. Upon reaching the ideal weight to each study, animals were randomly separated into different groups.

### 2.1. Dose-Response Curve and Toxicological Effects of MetfDeca

60 male Wistar rats of 300 to 320 g in weight underwent an intraperitoneally application of alloxan (150 mg/kg) and were monitored starting from the third day after administration of glucose and insulin (as indicated by the commercial kits). When hyperglycemia (HG) above 200 mg/dL was presented, the animals were appointed to the various working groups (*n* = 10) which were administered with doses of MetfDeca of 0.0, 0.7, 1.4, 2.8, 5, and 10 *μ*M. The oral doses were administered twice a week for a month; the dilutions were realized with sterile water. The glucose was measured once a week to establish hypoglycemic action of MetfDeca.

Once they completed the treatment, animals were sacrificed and serum was collected in BD Vacutainer® Venous Blood Collection system, centrifuged to 2500 r.p.m. during 5 min; glucose quantification was used for determination of a dose-response curve. Meanwhile, insulin, triglycerides, cholesterol, total bilirubin, aspartate aminotransferase (AST), alanine aminotransferase (ALT), *γ*-glutamyl transpeptidase (*γ*GT), alkaline phosphatase (ALP), sodium, potassium, urea, and creatinine assays were done as control of hypoglycemic, hypolipemic, and toxicological effects. For the toxicological effects, 10 intact rats of the same weight and age were used as negative control group (intact).

### 2.2. Effect of MetfDeca on Insulin-Independent Model (DM2)

80 male Wistar rats between 100 and 120 g were randomly divided into two groups: normal-calorie (NC) *n* = 20 and hypercaloric (HC) *n* = 60. The NC group was fed a balanced diet (Rodent Lab diet 5001) and the HC group was fed a diet high in calories (Patent: MX/E/2013/047377), which was designed with 71.4% carbohydrates, demonstrated by bromatological analysis. After 3 months of feeding with high-calorie diet, metabolically altered animals were validated. The validation was realized by body weight, abdominal perimeter and length from tip of nose to base of the tail, BMI (body mass index), body fat percentage, and serum parameters. Body weight of NC rats was 300 g, meanwhile HC rats reached 350–400 g [11].

The metabolically deregulated animals with HC diet were subdivided in 3 subgroups of *n* = 20, when these presented a minimal value of fasting glucose of 150 mg/dL and/or above 200 mg/dL in oral glucose tolerance test (OGTT, 1.75 g of glucose anhydrous/kg) and dyslipidemia characterized by hypertriglyceridemia and decline of high density lipoprotein cholesterol (HDL-C). In rats fed with high-calorie diet for 3 months, an oral glucose tolerance test (OGTT) was carried out after 4–6 h of fasting; an anhydrous glucose load of 1.75 g/kg was orally administered. Then glycemia was measured at 0, 30, 60, and 90 min by cardiac puncture previous anesthesia with ketamine + xylazine. OGTT was realized for the control group (diet NC, without treatment), group I-I (diet HC, without treatment), group I-I + metformin (diet HC, daily oral metformin 200 mg/kg), and group I-I + MetfDeca (diet HC, two times a week of MetfDeca 3.7 *μ*g/300 g (2.5 *μ*M) orally). Two months later, treatments ended and serum analysis was performed for HbA1c, OGTT, triglycerides, free fatty acids, cholesterol, and its fractions HDL-C, LDL-C, and VLDL-C. For which, animals were anesthetized (at a dose of ketamine + xylazine 0.2 mL/100 g intraperitoneally). 700 *μ*L of blood (approximately) was collected by intracardiac puncture with a BD Vacutainer Venous Blood Collection system and serum was separated for analysis. Immediately, rats were sacrificed and perfused with cold isotonic saline, and biopsies from tissues (liver, heart, kidney, and muscle) were taken and stored at −70°C for subsequent determination of glycogen and triglycerides according to established techniques [11].

### 2.3. Effect of MetfDeca on Insulin-Requiring Model (DM1)

60 male Wistar rats of 300 to 320 g in weight underwent a dose of 150 mg/kg of alloxan intraperitoneally. The model was validated when rats presented glucose levels above 300 mg/dL and hyperglycemia with hypoinsulinemia <5 *μ*U/mL. The groups were conformed (*n* = 20) in the next order: control group (without hyperglycemia), group I-R (hyperglycemia without treatment), group I-R + insulin (daily subcutaneous insulin, 2 IU per 100 mg/dL glucose), and group I-R + MetfDeca (7.18 *μ*g/300 g (4.8 *μ*M) of oral MetfDeca twice a week). Two months later, treatments ended. The insulin administered was a Humalog® Mix 75/25 (75% insulin lispro protamine suspension and 25% insulin lispro injection (rDNA origin)) which is a mixture of insulin lispro solution, a rapid-acting blood glucose-lowering agent, and insulin lispro protamine suspension, an intermediate-acting blood glucose-lowering agent. Two months later, treatments ended and serum analysis was performed. Similarly, to insulin-independent model (DM2), the extraction of serum and tissues was carried out. The serum and tissues extraction were carried out as previously described.

### 2.4. Biochemical Assays

The concentrations in serum of glucose, triglycerides, cholesterol, Low Density Lipoprotein-Cholesterol (LDL-C), Very Low Density Lipoprotein-Cholesterol (VLDL-C), High Density Lipoprotein-Cholesterol (HDL-C), total bilirubin, aspartate transaminase (AST), alanine aminotransferase (ALT), Gamma-glutamyl transpeptidase (GGT), alkaline phosphatase (ALP), urea, and creatinine in serum were determined with a semiautomatic analyzer BTS-350 (BioSystems). HbA1c analysis was performed using an I-Chroma Analyzer, which used immunofluorescence methodology. Electrolytes were measured in a Plus Lyte II analyzer of ion selective electrodes. Free fatty acid (FFA) concentration was determined according to the method described by Brunk and Swanson, 1981 [20]. Plasma insulin concentration was determined by an ELISA immunoassay (Diagnostica International Company), with the resulting antibody antigen complex assessed at 415 nm in a Stat fax 2600 plate reader (WinerLab Group). Insulin concentrations were obtained from a standard curve with a range of 0–200 *μ*UI/mL. The samples of oral glucose tolerance test (OGTT) were collected at the times 0, 30, 60, and 90 min after glucose load and stored in Eppendorf tubes. Samples were centrifuged at 8000 rpm for 10 min, and the serum was separated and frozen at −70°C until analysis. At the time of analysis, sera were thawed at room temperature.

### 2.5. Tissue Determinations

The biopsies from tissues (liver, heart, kidney, and adipose) were homogenized 100 mg in 800 *μ*L of isotonic saline solution (ISS) and proceeded to perform the protocol for the determination of glycogen by the technique described by Bennett et al. in 2007 [21]. Additionally, a second dilution was made for triglycerides content, in which the homogenate was diluted 1 : 2 with ISS and the protocol described by the manufacturer for the triglyceride kit was followed.

### 2.6. Statistical Analysis

Results were expressed as mean ± standard error of the mean (SEM). The results of toxicological effect model were evaluated and interpreted by statistical Student's “*t*” distribution at a significance level of *p* ≤ 0.05. Meanwhile, the biochemistry characterization and tissue measurements were evaluated by one-way ANOVA test considering *p* ≤ 0.05 significant, with Bonferroni post hoc test.

## 3. Results

**Figure 2:**
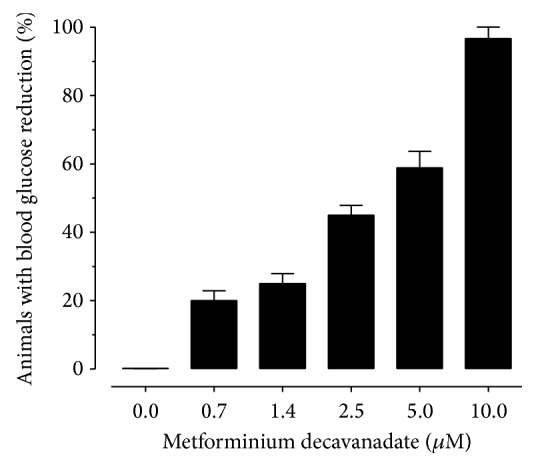
Administration of MetfDeca showing a lowering in blood glucose levels of hyperglycemic rats. The animals (*n* = 10/group) were administered with this compound at doses (0.7, 1.4, 2.5, 5, and 10 *μ*M) for 4 weeks in rats with alloxan-induced hyperglycemia (150 mg/kg). The graph shows the percentage of animals with reduced levels of glucose.

The administration of MetfDeca in hyperglycemic rats showed a decrease in blood glucose levels in dependence of the concentrations administered of compound. Quantitative analysis indicated that a concentration of 0.7 and 1.4 *μ*M of MetfDeca caused a reduction of glucose levels in the 20 and 25% of the animals, respectively. Likewise, the concentrations of 2.5, 5, and 10 *μ*M generated a reduction in serum glucose which corresponded to 45, 52, and 97%, respectively, as shown in Figure 2.

According to the results, the concentration of 5 *μ*M reduces glucose levels in 50% of subjects administered with MetfDeca. To perform the mathematical model which assigned the exact concentration of the compound that corresponded to effective dose 50 (ED_50_), we found that 4.8 *μ*M or 7.18 *μ*g/300 g is the adequate ED_50_. In this sense, the dose of 5 *μ*M for being the closest to ED_50_ was selected to assess the consequences in the metabolism of lipids and carbohydrates, as well as toxicological features.

Nonclinical studies for drug development according to European Medicine Agency include basic goals as identifying the pharmacological properties, in this particular case, glucose control and antilipemic features, and additionally understanding the toxicological profile as marked for the directive 2001/83/EC. Therefore, we carry out the basic toxicological profile of kidney and liver function, as well as triglycerides, cholesterol, and glucose-insulin.

The evaluation of features on metabolic control that MetfDeca possesses was assessed by the quantification of glucose that increases in rats with alloxan administration (150%); meanwhile, animals with alloxan and 5 *μ*M of compound recovered basal levels of the metabolite. With respect to insulin levels, rats with alloxan induction diminish in 86%. Rats with the compound did not show differences versus intact control. Triglycerides decrease in alloxan group and recovered to the normal levels in rats with administration of the compound. However, cholesterol did not show changes between groups (Table 1).

On the other hand, in Table 1, liver toxicological profile which is characterized by increases in enzymatic activity of AST, ALT, ALP, and *γ*GT was found in animals with only administration of alloxan, which similarly was the behavior of the levels of total bilirubin; all results were statistically significant *p* ≤ 0.05. When alloxan + 5.0 *μ*M of compound was analyzed, rats showed improvements on bilirubin levels, even below the control group, same case in AST and ALP activity with *p* < 0.05, while ALT remained significantly high (*p* ≤ 0.05).

Likewise, renal profile framed by urea, creatinine, sodium, and potassium concentrations were assessed (Table 1). Rat with alloxan increases significantly its levels of urea and creatinine in 25% and 86% more than control group; sodium and potassium decreased significantly in 9% and 25% as signs of altered renal function. Meanwhile, rats with additional treatment of the compound show recovery in the electrolyte balance (not entirely for sodium), but not in the nonprotein nitrogen levels which remain higher.

The preclinical development of a drug often includes pharmacology studies, in which the efficacy is assessed in appropriate animal models. In this study insulin-independent rats were used, as well as insulin-requiring rats to prove therapeutic efficiency of the MetfDeca. In order to justify the dosage used in clinical trials with models altered on lipids and carbohydrates.

**Figure 3:**
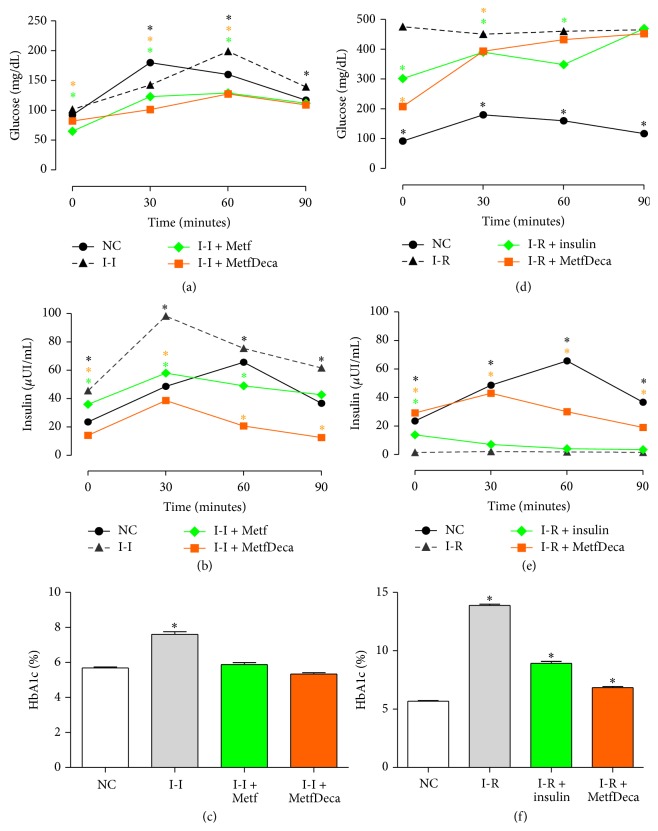
Oral glucose tolerance, insulin response, and fraction A1c of the glycosylated hemoglobin in different groups to two months of treatment. (a–c) Independent insulin (I-I) model. (d–f) Insulin-requiring (I-R) model. The results shown are the average of 5 different experiments ± SEM. *∗* indicates significant difference from the control group *p* ≤ 0.05 group by ANOVA test with a Bonferroni post hoc test.

Figure 3 shows results obtained in both models. In Figure 3(a) glucose tolerance in insulin-independent model with respect to the control group was demonstrated. The hypercaloric diet caused an increases in area under curve (AUC, dotted line) and a displacement to the right, characteristic of insulin resistance and metabolic syndrome. Both, the metformin and MetfDeca treatments diminished 23% and 27% the AUC, being significant to *p* ≤ 0.05 (AUC of NC = 4337; I-I + Metf = 3339; I-I + MetfDeca = 2182). Insulin (Figure 3(b)) in I-I group also showed an increase in AUC, which corresponded to 57% (AUC of I-I = 6815). The treatment with metformin returns to a normal secretion of the hormone, while treatment with MetfDeca reduces it until 50% of the AUC of the insulin secretion versus control group. Additionally, A1c fraction of the glycohemoglobin (Figure 3(c)) in the I-I group showed increase in 35% and a reduction without differences with respect to the control group in the animals with metformin and MetfDeca treatments.

Likewise, in insulin-requiring model evaluation of the handling of carbohydrate showed that I-R group increased in 300% of the AUC. The therapeutic efficiency of insulin and MetfDeca was compared with I-R group, while NC group only was used as reference of management therapeutic goal. Lispro insulin of rapid-acting and intermediate-acting was insufficient to control, because the AUC remained 250% above NC group but improved 18% with respect to I-R group (AUC of NC = 4337; I-R = 10842). The administration of MetfDeca observed similar behavior compared to insulin treatment (Figure 3(d)) (AUC of I-R + Metf = 8829; I-R + MetfDeca = 9075). Insulin concentration is showed in Figure 3(e), and I-R group presented very low levels of the hormone <2 *μ*UI/mL. Meanwhile, as it was expected insulin levels go down in the presence of glucose load until similar values to the I-R group. Insulin in MetfDeca group surprisingly presented normal levels of the hormone in fasting (19 *μ*UI/mL); however, the response does not achieve diminished plasmatic glucose; evidence suggests a recovery function of the *β* cells. This fact was corroborated by the HbA1c quantification; MetfDeca group showed better regulation with respect to the group with insulin treatment, and I-R group (Figure 3(f); 6.9%, 8.9%, and 13.9%, resp.).

**Figure 4:**
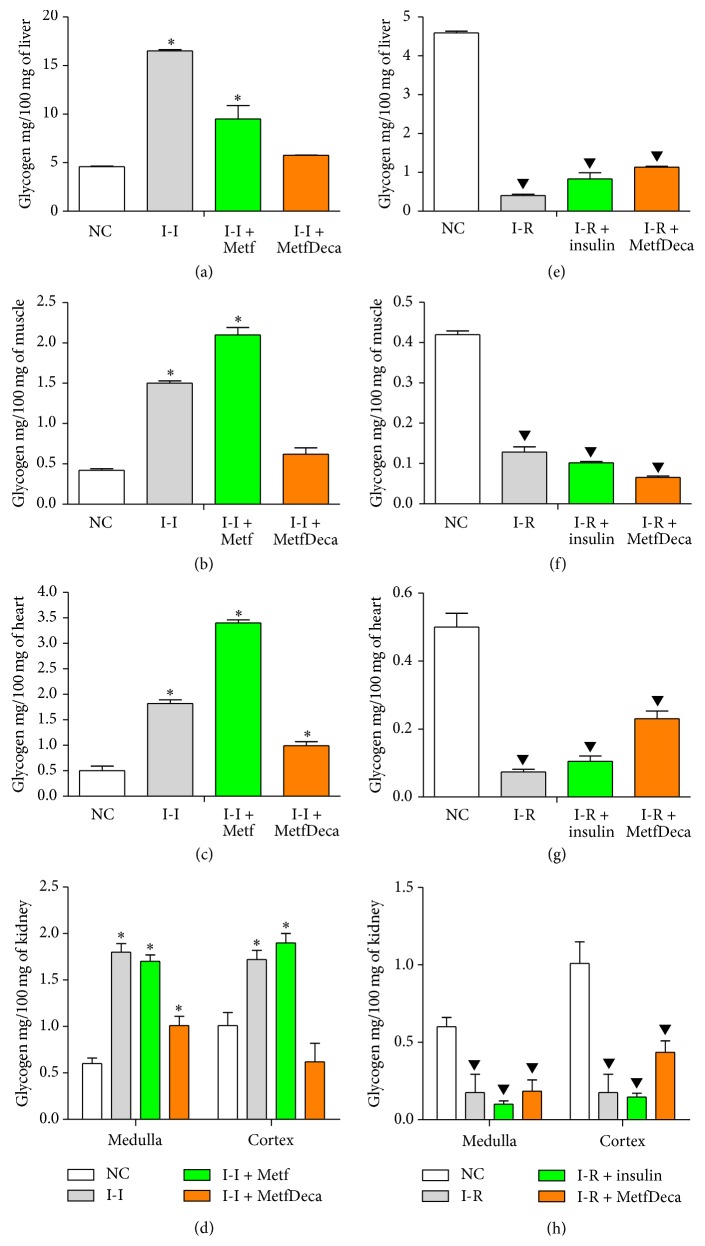
Glycogen content in different tissues from groups to two months with treatments. (a–d) Independent insulin (I-I) model. (e–h) Insulin-requiring (I-R) model. The results shown are the average of 20 different experiments ± SEM. *∗* indicates significant difference above from the control group (NC); meanwhile ▼ indicates significant difference below from the control group (NC), both with *p* ≤ 0.05 group by ANOVA test with a Bonferroni post hoc test.

The management of carbohydrates was confirmed by glycogen measurement in different tissues, in which recovery is indicative of effectiveness of the treatment. In this sense, insulin-independent model was evaluated; the animals fed with hypercaloric diet showed an increase of the glycogen stored in liver (259%), muscle (257%), heart (264%), renal cortex (70%), and renal medulla (200%). Metformin administration on other hand contrary to expectations does not reduce these levels, despite clear improvement of glucose levels in serum; glycogen levels in liver, muscle, heart, renal cortex, and medulla (107%, 400%, 580%, 88%, and 103%, resp.) were higher than NC group (significant, *p* < 0.05) Finally, treatment with MetfDeca showed better regulation on glycogen and management of carbohydrates, since in liver and muscle they remain 25% and 33% more in relation to NC, respectively. In renal cortex, glycogen content diminishes in 38%. Meanwhile, heart and renal medulla keep their glycogen levels significantly high in 98% and 68%, respectively; *p* < 0.05 (Figures 4(a)–4(d)).

In the DM1 model (I-R) a decrease of glycogen was observed, contrary to the one observed in DM2 model. In this case I-R group showed an important reduction of glycogen content, due to administration of alloxan which caused a depletion of serum insulin; glycogen formation is dependent on hormone signaling. Liver presented a significant reduction which correspond to 91%; muscle went down in 70%, and heart went down in 85%; and in kidney, the glycogen of the cortex diminishes 83%, while in medulla the decrement corresponded to 71%, all with statistic significant difference of *p* < 0.05. On the other hand, insulin treatment (daily subcutaneous insulin, 2 IU per 100 mg/dL glucose) which improved serum glucose management, however, in tissues did not show an important improvement; in liver and heart of I-R + insulin group with respect to I-R group, the glycogen stored increased 100% and 42%, respectively; however, muscle glycogen was not recovered and even remained 20% lower than I-R group. Kidney did not show differences versus I-R group. The group administered with MetfDeca improved with respect to I-R group in liver (178%), heart (213%), and renal cortex (147%). In renal medulla there are no changes regarding I-R group; meanwhile the glycogen in muscle was reduced the most with 50% below in the I-R group (Figures 4(e)–4(h)). Remarkably, in all treatments cases shown, a recovery regarding to NC group was not found.

The profile of serum lipids in which treatments were contrasted against intact animals (NC group) and impairment groups of DM2 (I-I) and DM1 (I-R) were measured with the goal to get to know the effect of MetfDeca. In the first section of Table 2 results of I-I model are shown that increased lipid profile in all parameters (TL 67%, TG 113%, Chol 37%, VLDL-C 28%, LDL-C 183%, and FFA 110%), except to HDL-C, which diminish 26.6%. When animals were treated with metformin the levels of total lipids, triglycerides, VLDL-C, LDL-C, and FFA were regulated; even HDL-C showed an increase of 26%; however, levels of cholesterol remained elevated slightly (23%); obviously, lipid profile improved considerably with regard to I-I group. Meanwhile, I-I + MetfDeca group normalized its concentration of total lipids, cholesterol, LDL-C, HDL-C, and FFA, but not in triglycerides concentration which remained increased in 70%, same case for VLDL-C with 39%; in relation to I-I group TL, cholesterol, LDL-C, and FFA showed a significantly reduction *p* ≤ 0.05.

In the DM1 model (I-R) we observed a complete lipid decompensation, classical of the diabetes. I-R group showed increases in TL (210%), triglycerides (467%), cholesterol (93%), VLDL-C (254%), HDL-C (87%), and FFA (360%); LDL-C did not show difference. Insulin treatment only reduced FFA levels, while the dyslipidemia remains present. Finally, MetfDeca treatment improved significantly lipid profile with respect to I-R group; however, TL maintained levels significantly above of NC group only in 27%, cholesterol in 51%, LDL-C in 271%, and FFA in 92%, as shown in Table 2.

**Figure 5:**
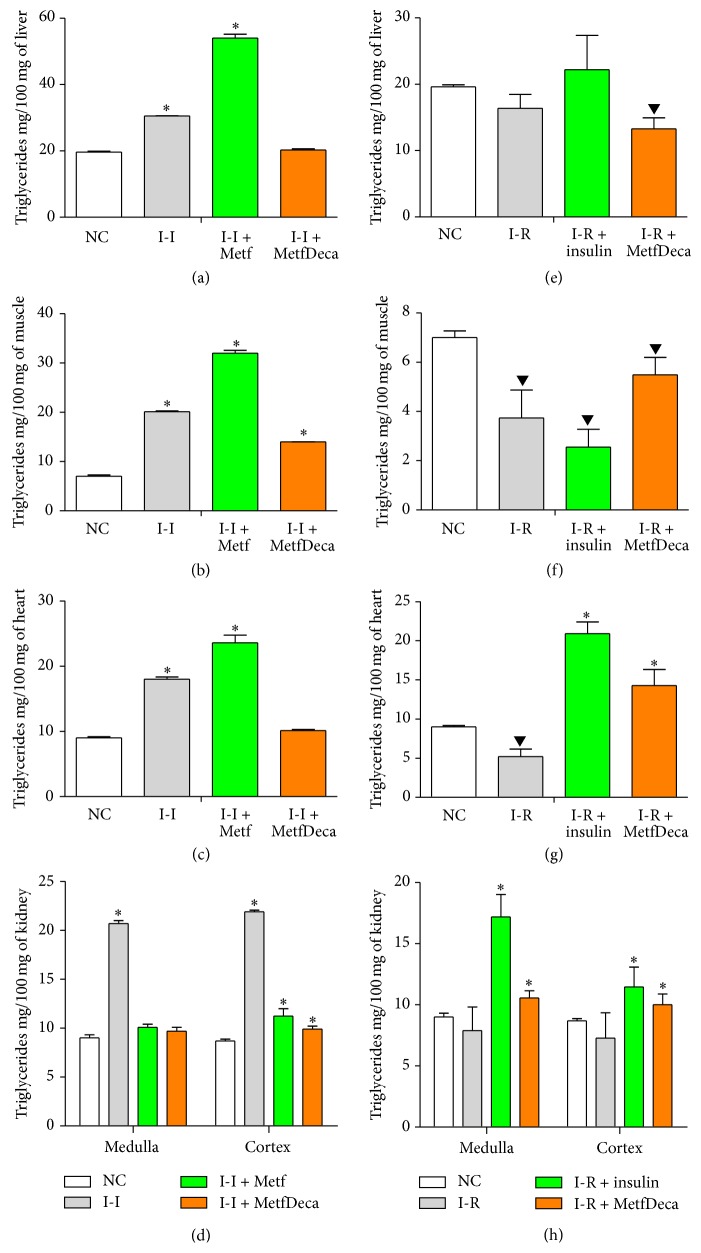
Triglyceride content in different tissues from groups to two months with treatments. (a–d) Independent insulin (I-I) model. (e–h) Insulin-requiring (I-R) model. The results shown are the average of 20 different experiments ± SEM. *∗* indicates significant difference above from the control group (NC); meanwhile ▼ indicates significant difference below from the control group (NC), both with *p* ≤ 0.05 group by ANOVA test with a Bonferroni post hoc test.

Pharmaceutical treatments most of the time are only limited to investigate serum effects; however, it is very important to consider the different tissues in relation to the metabolite analyzed. Therefore, triglycerides content in liver, muscle, heart, and kidney of experimental animals was also quantified as it is shown in Figure 5. In this sense, I-I group increases its triglycerides content in liver in 55.6%, muscle in 187%, heart in 130%, renal cortex in 130%, and renal medulla in 152%; these levels demonstrate multitissues steatosis. Metformin treatment only improves triglyceride levels in kidney which showed similar values to NC group. In liver, muscle, and heart metformin promoted a major increase of triglycerides of the 175%, 357%, and 162%. Meanwhile, with the MetfDeca treatment the distribution and levels of triglycerides in liver, heart, and kidney were regulated, although, in muscle which showed improvement this remained with a 100% more TG in relation to NC group. MetfDeca manages a better cellular lipid homeostasis than metformin treatment, which apparently produces a triglyceride redistribution toward other tissues.

In the animals administered with alloxan to get the insulin-requiring model, triglycerides levels were not affected in liver and kidney; however, in muscle and heart content showed a decrease of the 47% and 43%, respectively. Insulin treatment produced an overstoring in heart, renal cortex, and renal medulla from 132%, 29%, and 91%, respectively. While liver level remained unaffected and even in muscle went down more (63.5%) in relation to NC group. Meanwhile, MetfDeca treatment showed the lowest level of triglycerides in liver (32%), although in muscle the triglycerides only remained low in 22%. Contrary in heart, renal cortex, and renal medulla, content increased in 58.5%, 12%, and 17% above from NC group.

## 4. Discussion

In the guidelines to develop new medicinal products for treatment of diabetes mellitus specific strategies and steps should be considered, as pharmacodynamic data, in which the contribution to therapeutic and/or toxic effects should be discussed [22]. In this sense, the MetfDeca showed an important decrease of glycemia levels after 4 weeks of administration in the hyperglycemic alloxan-induced model, since decavanadate complexes have been described as insulin mimetics [6, 9–11, 23]. The most usual substances to induce diabetes in the rat are alloxan and streptozotocin (STZ) [24]. Despite the fact that STZ model is extensively recognized, this not only causes beta cell necrosis but also produces DNA alkylation in different tissues; cytotoxic effect is related to transport capacity of glucose through the facilitated glucose transporters GLUT2 and GLUT1 in pancreas, liver, kidney, and brain, mainly, producing cell death, structural and metabolic changes in these tissues, complicating the correct evaluation of therapeutic strategies [24–27]. Although the cytotoxic action of both STZ and alloxan is mediated by reactive oxygen species, the source of their generation is different. Alloxan and the product of its reduction, dialuric acid, establish a redox cycle with the formation of superoxide radicals. These radicals undergo dismutation to hydrogen peroxide and molecular oxygen. Thereafter highly reactive hydroxyl radicals are formed by the Fenton reaction. The action of reactive oxygen species with a simultaneous massive increase in cytosolic calcium concentration causes rapid destruction of *β* cells and could alter cells of different tissues; however the antioxidant defense prevents the severe injuries, so it is more suitable for studying therapeutic strategies on insulin-requiring diabetes mellitus [24].

As it was expected damage in exocrine pancreas was observed in rats with alloxan administration, because hyperglycemia with hypoinsulinemia was induced. It is important to emphasize that insulin signaling is associated with hepatic lipogenesis and mobility of triglycerides into VLDL; therefore, low levels of the hormone in DM1 early models produce lower levels of serum triglycerides, although these are accumulated in liver, as shown Table 1 [28–30]. Biochemical changes provoked by alloxan in blood have been associated with morphological and ultrastructural lesions in the liver that largely resembled chronic liver disease in humans linked to diabetes mellitus. Liver changes ranged from the fatty degeneration of liver cells to steatohepatitis and periportal fibrosis. Therefore, an increase in the hepatic enzymatic activity as has been reported in other studies exists, in which alloxan-induced diabetic animals increase blood levels of AST and ALT in the first 2 weeks after treatment, but only ALT remained significantly elevated until 26 weeks after diabetes induction [31, 32]. In this regard, we observed increased hepatic transaminases, *γ*GT, and ALP, as well as bilirubin, which by its levels could be interpreted as hepatic inflammation, but not as a toxic effect that derived in necrosis caused by alloxan administration.

On the other hand, kidney is the other organ involved in detoxification process and hidroelectrolitic homeostasis. Alloxan-induced diabetic rats showed increases of serum urea and creatinine classical in diabetic kidney, as well as loss of sodium and potassium, indicative of kidney function impairment [31, 33, 34]. Evidence suggests that hyperglycemia is associated with kidney damage since early stages, combined with an increase of reactive oxygen species and the resulting oxidative stress is thought to play a key role in the pathogenesis of this disorder [35]. Likewise, it has been demonstrated that alloxan causes both tubular and glomerular changes in structure, which imply a lost in the functions [33, 35]. In addition, a marked increase in the activity of Na^+^/K^+^-ATPase has been observed in the diabetic kidney probably because of an adaptation of nephrons to maintain electrolyte homeostasis in diabetes in face of the increased glomerular filtration rate (GFR) and osmotic diuresis [36].

After the conclusion that the dose with hypoglycemic feature of MetfDeca was 5 *μ*M/300 g/2 times a week, the metabolic response was evaluated, and surprisingly the fasting insulinemia level was normalized in the diabetic group. The insulinemia level of diabetic rats was normalized. Indeed, the decrease of blood glucose levels registered in diabetic rats exposed to vanadium compound may be related to the concomitant increase of plasma insulin concentrations. These results are in accordance with previous studies, in which insulin levels were recovered [37], fact attributable to the effectiveness of vanadium compounds [38, 39]. Protection on *β* cell could be due to antioxidant feature that has been described, in which antioxidant stress markers as superoxide dismutase, catalase, and glutathione peroxidase are dependent on the nature of the polyoxovanadates present, besides the concentration administered, because higher levels of decavanadate can produce an increase of superoxide anion and cellular damage associated with Fenton-like reactions as it is observed in transitional elements [6, 23]. Taking advantage of the minimal concentration of decavanadate administered, cells with vanadium uptake can improve oxidation of energetic molecules such as glucose and lipids and through insulinemia restarted the conversion of these energetic molecules in energetic stored triglycerides, which are mobilized into the bloodstream. Liver is the main organ involved in this mechanism, our results showed that, in rats treated with MetfDeca for one month, liver recovered almost totally its enzymatic activity, probably associated with improvement in metabolic conditions and reduction of oxidative stress, with exception of ALT that remain increased. ALT levels are in concordance with urea behavior because this transaminase is involved in the transferring of NH_3_^+^ groups to *α*-ketoglutarate to form glutamate which take part in gluconeogenesis, generating urea as final product. Uremia is considered a kidney damage marker; however, in conditions of sodium loss, Henle's loop used urea as countercurrent osmotic gradient to achieve sodium recovery [40, 41]; for this reason rats administered with MetfDeca improve sodium and potassium levels, but also creatinine serum almost achieved normal values. Pharmacodynamical data obtained have showed that contribution to therapeutic effect of MetfDeca could be appropriated, because toxic effects are to some extent negligible.

Insulin-mimetic attributes of decavanadate were challenged in two models with different metabolic disturbances, but with hyperglycemia in common. Usually, only the serum biochemical behavior of glucose and lipids is considered but does not take into account the intracellular behavior of these molecules, which are controlled by insulin signaling; thus, when we talk about insulin-mimetic activity, these actions should be considered to clarify the limitations of vanadium compounds.

First, an insulin-independent model was developed, which possesses clinical phenotype of glycemic response impaired, A1c glycohemoglobin elevated, and dyslipidemia with hypertriglyceridemia and cholesterolemia (VLDL and LDL fractions elevated with HDL decrement), despite hyperinsulinemia present as it happens in type 2 diabetes mellitus. Additionally, classical complications by insulin signaling also were presented: increases in glycogen levels were observed in liver, muscle, heart, and kidney in cortex and medulla zones, and steatosis in the evaluated tissues (triglycerides stored) was also observed.

American Diabetes Association as well as European Association for the Study of Diabetes recommended metformin monotherapy as initial pharmacological treatment in type 2 diabetes mellitus, due to the fact that it has a long-standing evidence base for efficacy and safety, is inexpensive, and may reduce risk of cardiovascular events; position statement evaluated the data and developed recommendations, including advantages and disadvantages, for antihyperglycemic agents for type 2 diabetic patients [13]. However, its study is based mainly on serum evidences and not on tissues behavior. Previously, we reported risk of developing lactic acidosis and alterations in multiple tissues in triglycerides and glycogen storages [11]. In this work, we reproduced independent insulin model in Wistar rats that were treated with metformin monotherapy following pharmacological recommendations of ADA. The subjects studied observed regulation on glucose tolerance, insulin secretion, A1c glycohemoglobin fraction, and dyslipidemia. However, intracellular glycogen in liver, muscle, heart, and kidney were not improved, same case to triglycerides stored in these tissues; this last phenomenon is recognized as steatosis and is common in excess of metabolic needs. Steatosis can generate lipotoxicity by lipotoxic intermediates such as ceramide and acylcarnitine. Collectively these events favor oxidative stress and apoptosis, and mitochondria become damaged further compromising ATP production; lipotoxicity is related also to endoplasmic reticulum stress through mechanisms related to oxidative stress and mitochondrial dysfunction and by inducing an inflammatory response [42, 43].

Moreover, other groups with same independent insulin conditions were treated with pharmacological monotherapy of MetfDeca 3.7 *μ*g/300 g (2.5 *μ*M) two times a week, a dosage of 48,000 times less than metformin. In these studies the subjects also observed improvement on glucose tolerance and regulation in insulin secretion with A1c fraction of glycohemoglobin normalized, despite the fact that they remain fed with HC diet. Glycogen stored is showed to be regulated in almost all tissues that were evaluated; minor increases remain in heart and kidney medulla. Likewise, dyslipidemia profile was improvement, although triglycerides did not reach values as normocaloric group. However, steatosis was removed in all tissues evaluated, and minimal levels remain in muscle and kidney cortex, but these do not have biological impact, because they mean a recovery of insulin resistance previously generated by HC consumption [44]. Our results showed that decavanadate in combination with metformin has a biological activity, although the exact mechanism is unknown completely; evidence has demonstrated an improvement in insulin signaling, as in the case of protein kinase B (PKB) that favors the regulation of signals with subsequent inhibition of lipolysis; this argument is supported in inhibition of protein tyrosine phosphatase 1B (PTP 1B); this enzyme provides a negative feedback by catalyzing the dephosphorylation of the insulin receptors. The dephosphorylation of the insulin receptor slows the intake of the glucose from the blood by not allowing the other proteins in the insulin transduction pathway to be activated and consequently not to do their job of transferring the signal to the other proteins in the pathway, so it favored the correct insulin signaling [45–47]. Pharmacological action of decavanadate and metformin in low dosage which can act simultaneously in different tissues and organs favoring the oxidation and burning of carbohydrates and lipids in a more regulated way thus has an advantage over the counter ion alone as monotherapy [11]. However, this model has the presence of insulin, so it could not know if MetfDeca is an insulin-mimetic agent or only improves the cellular environment and then insulin present does its work.

In order to investigate insulin-mimetics effects of decavanadate we develop a model insulin-requiring by alloxan injection in which observed insulin depletion with hyperglycemia that produces an increase of glycohemoglobin occurs in type 1 diabetes mellitus. Normally, when insulin binds to its receptor, it activates the glycogen synthesis by inhibiting the enzymes that slow down the PI3K pathway such as PKA enzyme. At the same time, it will promote the function of the enzymes that provide a positive feedback for the pathway like the AKT. The inactivating enzymes that stop the reaction and activating enzymes that provide a positive feedback will increase glycogen, lipid, and protein synthesis, therefore promoting the normal glucose intake. Therefore, if insulin signaling is missing, glycogen synthesis is diminished as shown in Figures 4(e)–4(h). Additionally, DM1 usually has a tendency for the late development of dyslipidemia associated with cardiovascular risk factors and microvascular complications [48–51]. Both dyslipidemia and impairment of stored triglycerides were observed in this study; mostly effect was presented by muscle and heart (Table 2 and Figures 5(e)–5(h)). Thus, DM1 model was validated as suggested by the guidelines to pharmaceutical development.

Recommended therapy for type 1 diabetes by international associations consists of initiation and management of insulin therapy with insulin analogs to achieve desired glycemic goals [13]. Following the guide, the initiation and management of insulin therapy in rats of the DM1 model were administered daily subcutaneous insulin, 2 IU per each 100 mg/dL glycemia. Insulin administered was a Humalog Mix 75/25 (rDNA origin) that is a mixture of insulin of rapid-acting (25%) and insulin lispro protamine suspension that acts as an intermediate agent. Glycemia with insulin treatment was recovered gradually; however, therapy apparently was good but insufficient because fasting glucose did not return to appropriate levels; furthermore, basal insulin levels also remain diminished, and in consequence, DM1 model did not recover its glucose tolerance, not even complete regulation in A1c glycohemoglobin levels; same case occurs for lipids profile in which there are improved parameters, but not to required levels. When these situations occur the guide recommended three injections of rapid-acting insulin analog administered just before eating or regular human insulin premixed formulations (70/30) that are rapid-acting insulin analogs, but their pharmacodynamic profiles are suboptimal for the coverage of postprandial glucose and its monitoring must be monitoring completely [13]; for this reason in an animal model it is difficult to be implemented; however, adjustments were made in insulin administered dosage based on the prevailing blood glucose levels in each animal, until understanding of the pharmacodynamic profile of formulation Humalog Mix 75/25. After glycogen and triglycerides in tissues were quantified, data showed minimal increase of glycogen levels that correspond to a cellular oxidative dynamic appropriate in which reserves of carbohydrates and triglycerides are employed to obtain energy [52]. In this regard, muscle presented the features aforementioned; however, heart and kidney presented an overstored triglycerides, precisely in relation to low capacity of energetic spend promoted by insulin administration, in contrast with liver which had no difference between groups. This apparent discordance between ideas is in relation to insulin which stimulates intracellular triglyceride synthesis while inhibiting lipolysis in tissues with low energy obtained by lipids [53–55].

Pharmacological dosage of MetfDeca administered in DM1 model showed an improvement in glucose tolerance and insulin secretion, in fact, rats previously insulin-depleted showed an important secretion of the hormone after MetfDeca administration, in accordance with other study, suggesting a recovery of insulin secretion ability by Langerhans islets, as well as an insulinotropic property of the vanadium compound studied here [37]. Previously, processes of beta cells regeneration from extraislet precursor cells in mouse model induced by selective perfusion of alloxan have been reported [54, 55]. Indeed, in our DM1 model, MetfDeca treatment might stimulate beta cell proliferation from intraislet endocrine cells, as well as differentiation from extraislet precursor cells. Therefore, MetfDeca promoted a best reduction in A1c fraction of glycohemoglobin that corroborated beneficial actions of MetfDeca. Vanadium compounds have shown that they can influence glucose and lipid metabolism by insulin-dependent or insulin-independent biochemical pathways [56, 57]. Insulin-independent mechanism of vanadium compounds is mediated through activation of PKB/AKT kinase leading to the glucose uptake by the GLUT4 transporter [58, 59]. Also, activation of PKB/AKT stimulates the phosphorylation of GSK3, resulting in the stimulation of glycogen synthesis [57, 60]. However, we observed a partial glycogen restauration, but better than insulin treatment.

In relation to lipids profile MetfDeca improved all parameters measured, even better than insulin treatment, although it was not enough to resemble the intact control group. Lipids behavior suggests an improvement in tissues in relation to energy obtaining mode, because rates of hepatic triglyceride synthesis from fatty acid esterification are dependent on substrate flux and independent of circulating plasma insulin concentrations; thus, when serum FFA diminish liver lost flux of prime matter to build triglycerides, results suggest strongly that MetfDeca induced lipidic burning, as in DM2 model [61, 62]. This last idea was supported by the triglyceride content in tissues; these showed a subtle regulation in each tissue evaluated. Apparently, decavanadate could induce the formation of ATPase dimers, eventually relevant to ATPase activity, and at same time can interact with mitochondrial complex III, limiting electron chain which diminishes ATP formation so that energy obtaining would be by lipid burning (*β*-oxidation); the induction of the production of ROS would be expected [6, 7, 9, 46].

## 5. Conclusion

A potential metallopharmaceutical MetfDeca has been developed and in this study it has been shown to be a good alternative that could be considered as adjuvant in the treatment of diseases that presented hyperglycemia and dyslipidemia, because low concentrations do not generate toxicity conditions and it is very efficient as hypoglycemic and hypolipidemic agent due to properly redistributing the excess of these molecules to different organs in a similar way to insulin. However, although MetfDeca has been considered as an insulin-mimetic agent, actually it acts as an insulin-enhancer agent in many aspects, ranging from protection to the possible generation of beta cells, but highlighted its intracellular mechanisms in the management of lipids. Therefore, pharmaceutical goals as pharmacodynamic data and therapeutic contributions as well as toxic effects have been analyzed. Next step should be the precise description of the action mechanism. Work is in progress in this regard.

## Figures and Tables

**Table 1:** Metabolite control, renal and hepatic toxicological profile with dose of 5.0 *μ*M MetfDeca.

Metabolite	Control	Alloxan + 0.0 *μ*M (MetfDeca)	Alloxan + 5.0 *μ*M (MetfDeca)
Insulin (*μ*U/mL)	25.1 ± 5.2	3.4 ± 0.3▼	23.6 ± 3.6
Glucose (mg/dL)	122.4 ± 2.7	300 ± 13.7▲	129 ± 4.6
Triglycerides (mg/dL)	96.3 ± 5.1	55 ± 3.4▼	138 ± 9.6▲
Cholesterol (mg/dL)	62.1 ± 2.4	58 ± 1.8	65 ± 3.6
Total bilirubin (mg/dL)	0.7 ± 0.1	0.95 ± 0.1▲	0.51 ± 0.25
AST (U/L)	230 ± 16	316.5 ± 2.3▲	117.9 ± 3.1▼
ALT (U/L)	60 ± 1.65	86.4 ± 2.5▲	91.1 ± 1.2▲
*γ*GT (U/L)	7.3 ± 0.75	14.6 ± 0.29▲	8.7 ± 0.21
ALP (U/L)	316 ± 23	620.9 ± 4.9▲	267.3 ± 8.1▼
Sodium (mmol/L)	144 ± 0.1	131.2 ± 0.5▼	137.5 ± 1.5▼
Potassium (mmol/L)	6.6 ± 0.1	4.91 ± 0.7▼	6.8 ± 0.4
Urea	36 ± 0.41	45 ± 3.6▲	67 ± 2.3▲
Creatinine	0.53 ± 0.01	0.96 ± 0.1▲	0.8 ± 0.09▲

The serum parameters are the average of 10 separate experimental animals ± SEM. ▲ indicates significant difference with values above the control group, while ▼ indicates significant difference with values below the control group with *p* ≤ 0.05 by Student's *t*-test. AST: aspartate transaminase, ALT: alanine aminotransferase, *γ*GT: Gamma-glutamyl transpeptidase, and ALP: alkaline phosphatase.

**Table 2:** Serum lipids in independent insulin model and insulin-requiring model with pharmacological treatments and MetfDeca.

Metabolite	NC (*n* = 20)	I-I (*n* = 20)	I-I + Metf (*n* = 20)	I-I + MetfDeca (*n* = 20)	I-R (*n* = 20)	I-R + insulin (*n* = 20)	I-R + MetfDeca (*n* = 20)
Total lipids (mg/dL)	176 ± 11	294 ± 20▲	190 ± 12**↓**	203 ± 20**↓**	546 ± 28▲	502 ± 21▲	223 ± 16▲**↓**
Triglycerides (mg/dL)	63.5 ± 5.0	135 ± 13▲	68 ± 6.2**↓**	108 ± 5▲	360 ± 10▲	340 ± 13▲	75 ± 3**↓**
Cholesterol (mg/dL)	89.5 ± 2.4	123 ± 14▲	110 ± 6.9▲	87 ± 3**↓**	173 ± 8▲	145 ± 18▲	135 ± 11▲**↓**
VLDL-C (mg/dL)	16.5 ± 1.8	21.2 ± 2.0▲	20 ± 1.5	23 ± 2▲	58.4 ± 2▲	29.1 ± 1.3▲**↓**	17 ± 2**↓**
LDL-C (mg/dL)	23 ± 2.6	65.1 ± 2.8▲	26.9 ± 2.5**↓**	24 ± 3**↓**	21 ± 1.8	31.4 ± 3▲	73 ± 5▲
HDL-C (mg/dL)	50 ± 6.5	36.7 ± 1.1▼	63.1 ± 3.2▲	40 ± 5	93.6 ± 11▲	84.5 ± 6▲	45 ± 2.4**↓**
FFA (mg/dL)	2.5 ± 0.6	5.5 ± 1.2▲	2.8 ± 0.4**↓**	2.3 ± 0.2**↓**	11.5 ± 1.2▲	3.2 ± 0.3**↓**	4.8 ± 0.8▲**↓**

The results shown are the average of 20 separate experimental animals ± SEM. ▲ indicates significant difference with values above the control group with normal calorie diet, ▼ indicates significant difference with values below the control group with normal calorie diet, while **↓** indicates significant difference with respect to groups I-I and I-R, *p* ≤ 0.05 one-way ANOVA test with Bonferroni post hoc test.
